# New microsatellite markers for assessment of genetic diversity in date palm (*Phoenix dactylifera* L.)

**DOI:** 10.1007/s13205-011-0010-z

**Published:** 2011-05-27

**Authors:** Khaled Elmeer, Hina Sarwath, Joel Malek, Michael Baum, Aladdin Hamwieh

**Affiliations:** 1 Biotechnology Centre, Ministry of Environment, Doha, Qatar; 2 Weill Cornell Medical College in Qatar, Doha, Qatar; 3 International Center for Agricultural Research in the Dry Areas (ICARDA), Aleppo, Syria

**Keywords:** *Phoenix dactylifera*, Microsatellite marker, Simple sequence repeats

## Abstract

New primer pairs of genomic DNA microsatellite markers were tested to assess the genetic diversity of eleven date palm genotypes. The results indicated that out of thirty, only seven primers (23.3%) failed to amplify the expected PCR fragments, while thirteen primers (43.3%) amplified monomorphic banding patterns and the remaining ten primers (33.4%) generated polymorphic banding patterns. A total of 77 alleles have been observed with a mean of 7.7 alleles per locus. The average of gene diversity was 0.80 ranging from 0.6 (in marker DP168) to 0.9 (in two markers DP157 and DP175). These new co-dominant markers will be a starting point for researchers making use of the markers for genetic mapping and diversity analysis of date palm.

## Introduction

Date palm (*Phoenix dactylifera* L.) belonging to the *Arecaceae* family, originated in Mesopotamia and thousands of cultivars have been reported (Hanachi et al. 1998). Date palms is a diploid (2*n* = 2*x* = 36), and the predicted genome size is estimated to be approximately between 550 and 650 Mbp long (Malek 2010). Date palms have always been clonally propagated to ensure the identity and uniformity of the cultivars and the morphological markers of many traits are often unreliable or extremely difficult to be estimated correctly, especially because of the influence of environmental factors (Elhoumaizi et al. 2002). Therefore, marker technology for DNA fingerprinting has become increasingly important in recent years to discriminate among closely related cultivars. Several marker systems have been used to study the genetic diversity of date palm. In brief, randomly amplified polymorphic DNA (RAPD) fingerprints have been used to identify date palm accessions in Algeria (Benkhalifa 1999), in Morocco (Sedra et al. 1998), in Tunisia (Trifi et al. 2000), in Saudi Arabia (Al-Khalifah and Askari 2003), and in Egypt (Soliman et al. 2003; Adawy et al. 2006). Amplified fragment length polymorphic (AFLP) markers have been applied to study the genetic diversity of date palm cultivars in Egypt and California (Cao and Chao 2002; El-Assar et al. 2005; Adawy et al. 2006).

Microsatellite or simple sequence repeat (SSR) marker has been used in plant diversity analysis; the popularity of these markers is due to their ease of amplification by polymerase chain reaction (PCR), their co-dominant nature and their typically high levels of allelic diversity at different loci. There are numerous reports suggesting the usefulness of microsatellite markers for measuring the genetic variability in a wider taxonomic range (Ryberg et al. 2002; Li et al. 2007; Kawka et al. 2007; Chan et al. 2008; Banhos et al. 2008).

Microsatellite markers have been developed and used to investigate genetic diversity in *Phoenix dactylifera* (Billotte et al. 2004). They used (GA)*n* microsatellite-enriched library to develop 16 microsatellite markers. More recently further 17 microsatellite loci were developed by constructing two microsatellite-enriched libraries of date palm using (GA)*n* and (GT)*n* repeats (Akkak et al. 2009). These microsatellite markers have been used to assess the genetic diversity and relationships of date palm varieties in Tunisia (Zehdi et al. 2004), in Sudan (Elshibli and Korpelainen, 2007), in Oman (Al-Ruqaishi et al. 2008), and in Qatar (Ahmed and Al-Qaradawi 2009). However, still 33 microsatellite markers are not enough to cover the whole genome and give a comprehensive assessment of biodiversity in date palm. This creates an urgent need to develop more of microsatellite markers in this genome. The objective of this research is to use recently developed new microsatellite markers in date palm genome to assess the genetic diversity in date palm.

## Materials and methods

### DNA extraction

Eleven cultivars were chosen randomly from different locations in Qatar namely Khalas, Shaishi, Barhi, Hilali, Sukari, Khnaizi, Shahil, Khasab, Rzaiz and Lulu as females and one male Phahel.

The frozen young leaf tissues of date palm collected from each of the cultivars were first cleaned carefully with distilled water to remove the waxy layer and then 1 g of leaf sample was cut into small pieces and grinded into fine powder using liquid nitrogen. DNeasy Plant Maxi kit protocol (QIAGEN) was used to extract DNA by following the manual instructions of the kit (DNeasy Plant Handbook). The obtained DNA were quantified and qualified using Nanodrop Spectrophotometer. For further estimation of the DNA quantity 2 μl was loaded on 0.85% agarose gel at 100 V for 30 min. The gels were stained in ethidium bromide and visualized under UV light.

### Primer design and PCR amplification

The sequences of 30 new primer pairs of genomic DNA of date palm were obtained from the International Center for Agricultural Research in the Dry Areas (ICARDA) recently published by Hamwieh et al. (2010). These primers were made in (IDT) Integrated DNA Technologies, Inc and derived from an assembly draft of the date palm genome generated by whole genome shotgun next generation DNA sequencing issued by researchers in the Weill Cornell Medical College in Qatar (WCMC-Q).The length of the primers, sequence information, repeat motifs, allele size and melting temperature (*T*_m_) are shown in (Table 1). Polymerase chain reaction was performed in a total reaction mixture of 25 μl containing 2 μl (20–30 ng) of total genomic DNA, 12.5 μl of AmpliTaq Gold^®^ 360 Mastermix (Applied Biosystems), 1 μl (5 pmol/μl) of primers each and 8.5 μl of nuclease free water. Amplification was carried out in a Veriti 96 Well Fast Thermal cycler (Applied Biosystems) under the following conditions: initial denaturation 95 °C for 10 min, 35 cycles (denaturation 95 °C for 30 s, annealing  temperature depending on primer for 30 s, extension 72 °C for 1 min), final extension 72 °C for 7 min.

**Table 1:** Forward and reverse primer sequences, repeat motifs and expected sizes of microsatellite loci and its status of amplification of SSR loci of date palm

Primer name		Primer’s sequence	*T*_m_ (°)	Expected size	Motif repeat	Status of amplification
DP150	F	CTGCGCCAATCTAAACCATT	52.6	177	(GAA)9	−
	R	GCAAATTGCAACAAATCCTTG				
DP151	F	TTGCTGGTTGAAATGGTGTT	53.3	168–186	(AC)37	++
	R	GCAACAGATGCTCTTGCTCA				
DP152	F	ACGAGTTTTTGGGAGAGCAA	53.6	224	(TAT)8	−
	R	GCAAGTTGCCAACATTCTTGT				
DP153	F	TCATCACAGGCAATGGCTAA	51.6	204	(TCA)9	+
	R	GCAGATGGCCATTGAACC				
DP154	F	ACACACACACACCGCGAAT	53.1	249	(AC)19	+
	R	GCATGTGAGGCGCATATCTA				
DP155	F	CCCCCTCTCTCTCTCTCTCTC	55.8	200	(TC)51	−
	R	GCCAAGAGGATTGGAGATTG				
DP156	F	TGTGTGTGTGTGTGTGTGTGA	54.6	221	(GA)17	+
	R	GCCATTGTTTGTGTGGACTG				
DP157	F	TGGACAATGACACCCCTTTT	54.6	180–244	(TC)19	++
	R	GCCCACACAACAACCTCTCT				
DP158	F	TCATTGGCTAATCCACACACA	54.3	204	(GA)29	−
	R	GCCTTGTGGTCATGAGAGGT				
DP159	F	AGCTCCAATTTGCTGCAGAG	54.3	156–172	(TC)27	++
	R	GCTGACCTGGAGTCCAAAAC				
DP160	F	AAGAGCGACAATCATGACCA	57.7	108–136	(GAAA)5	++
	R	GGAAATTGAAGGGCATCTTG				
DP161	F	TGGTTGCTGCTTATCTGCTG	54.9	211	(CT)13	+
	R	GGAGGGAACCGAGAGAGAGA				
DP162	F	TGGACTTCAAGAAGTGCGAAT	57.3	183	(TACA)9	−
	R	GGCAGTCACATTTTGCTTCA				
DP163	F	GTGCGTGTGTGTGTGTGTGT	53.4	215	(GA)19	+
	R	GGCTGTTTGGGTTCGTACTG				
DP164	F	GGACCAAGAACCGACAGTTG	57.1	200	(ATAG)6	+
	R	GGGAAGGTGAGGTGGTATGA				
DP165	F	AAGCATCCTATGGCTTTGACA	54.8	222	(AATA)5	+
	R	GGGCTGTATGTGATGCATTG				
DP166	F	CAATTTCTTCTCGCCTGGAG	54.5	210	(GAAA)5	+
	R	GGGGTTTCTTTTCCTTCTGC				
DP167	F	ACATCCAATGGCATCCAAAT	57.6	243	(GAAA)6	+
	R	GGGTTTCCAGGTTTTCTTCTC				
DP168	F	GCAGCAAAGCCCTTAGGC	54.3	163–175	(CAT)8	++
	R	GGTGTTATGTGCAGCCAATG				
DP169	F	GCATGGACTTAATGCTGGGTA	57.1	129–223	(AAT)12	++
	R	GGTTTTCCTGCCAACAACAT				
DP170	F	TCTTTGGGCTTACGACAACC	55.9	195–227	(AGGG)5	++
	R	GTATGGCCCAAGATGCAGAT				
DP171	F	GTGGGAGTAGCGAGGTATGG	56	197–218	(TTC)10	++
	R	GTCCGGCACTTTAGGAAGTT				
DP172	F	GGTGTTTGGGCCTATTTCCT	54.2	199–235	(AGG)11	++
	R	GTCCTCCTCCTCCTCTGTCC				
DP173	F	CCACATGCAATTTCCAAAAG	51.9	198	(TC)27	+
	R	GTGCGTATCGGGAGAGAGAG				
DP174	F	CTCTGTCGTACGGAGGAAGG	55.6	187	(CGTG)5	−
	R	GTGGCACTATCACGCTCTCA				
DP175	F	ACACACACACACACACACACC	57.6	196–274	(CA)19	++
	R	GTGGCTTCTTTTTGGCTGTC				
DP176	F	GCCATTAACGAAATGGCTTG	54.5	198	(CAA)9	+
	R	GTTTGCACATAGCGCTTCAA				
DP177	F	TTCCTTGGGCTCACTTCAAC	54	216	(AGGC)6	+
	R	TAACATGCCAGCAAAGGTGA				
DP178	F	AGTTTGTCAGGCCATTTGGT	58.7	186	(TC)19	+
	R	TACATGTGCGTATCGGGAGA				
DP179	F	GGTTAGCCATCCAAAAGTGG	56.2	183	(ATTT)5	−
	R	TATGTAGCCTCCACCGCATC				

−, Non-amplification; +, amplification of monomorphic band; ++, amplification of polymorphic band of desired PCR product with Qatari date palm

In order to achieve better resolution, electrophoresis was performed using the Spreadex^®^ EL600 Mini gel (Elchrom Scientific). These gels are manufactured from a novel monomer and have 3× higher resolving power than any other synthetic gels with working separation range 40–600 bp and optimal separation range 150–350 bp. The amplified DNA fragments 2 μl were diluted with 3 μl distilled water and 2 μl of loading dye making a total volume of 7 μl were loaded on to the gel. Electrophoresis was carried out at 150 V for 150 min in 1× TAE buffer (30 mM). The gel was stained with ethidium bromide (EtBr) solution (1 mg/ml) for 30 min at 350 rpm and destained with double distilled water for 45 min at 400 rpm. The DNA banding patterns were visualized on a UV transilluminator and documented using Gel Documentation System (Alpha Innotech).

*Data analysis.* Microsatellite bands were precisely measured by gel documentation system AlphImager EC by Alpha View software V.3.0.0.0 and scored for each genotype. Each polymorphic DNA band at particular position on the gel was treated as a separate character and scored as allele size. Data were then computed with the PowerMarker software V3.0 (Liu and Muse 2005) to detect the percentage of heterozygosity and the phylogenetic relationship among the genotypes on the basis of the allele’s size. The phylogenetic diagram was drawn by TreeView V.1.6.6 (Page 1996).

## Results and discussion

**Fig. 1:**
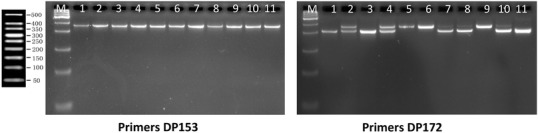
Markers amplified monomorphic and polymorphic banding patterns at expected size: *M* is 50 bp Genplot marker, date palm sample Cultivar No. *1* Khakas, *2* Shaishi, *3* Barhi, *4* Hilali, *5* Sukari, *6* Khanaizi, *7* Shahil, *8* Khasab, *9* Razaiz, *10* Lulu and *11* Male

Thirty new primer pairs of genomic DNA microsatellite markers of date palm were tested to assess the genetic diversity of eleven date palm genotypes. The results indicate that ten of the thirty markers (33.4%) generate polymorphic banding patterns at expected size (Fig. 1). These primers are: DP151, DP157, DP159, DP160, DP168, DP169, DP170, DP171, DP172 and DP175 (Table 1). These new co-dominant markers will be a starting point for researchers making use of the markers for genetic mapping and diversity analysis of date palm. However, these results are relatively lower when compared to Akkak et al. (2009), who selected seventeen markers (41%) as polymorphic, after screening of forty-one simple sequence repeats. The results also show that thirteen markers (43.3%) amplified monomorphic banding patterns (Fig. 1), while the remaining seven primers (23.3%) failed to amplify the expected PCR fragments.

The microsatellites examined were highly polymorphic possessing a great number of alleles, a total of 77 alleles with a mean of 7.7 alleles per locus were scored, however, the number of alleles varied between 4 in primer DP168 and 12 in primers DP157 and DP175 (Table 2). The number of alleles per locus detected in this study were higher than those scored by Ahmed and Al-Qaradawi (2009) who marked 40 different alleles with a mean of 4 alleles per locus by examining 15 Qatari date palm cultivars.

**Table 2:** Genetic biodiversity information of Qatari date palm using 10 SSR markers

Marker	Major allele frequency	Genotype no.	Allele no.	Gene diversity	Heterozygosity	PIC
DP151	0.36	7.00	7.00	0.79	0.00	0.77
DP157	0.18	9.00	12.00	0.90	0.45	0.89
DP159	0.36	7.00	6.00	0.78	0.27	0.75
DP160	0.27	9.00	8.00	0.84	0.45	0.82
DP168	0.59	4.00	4.00	0.60	0.36	0.55
DP169	0.31	8.00	8.00	0.80	0.36	0.77
DP170	0.27	8.00	7.00	0.80	0.91	0.79
DP171	0.36	8.00	7.00	0.79	0.45	0.76
DP172	0.36	8.00	6.00	0.77	0.18	0.74
DP175	0.13	10.00	12.00	0.90	0.73	0.89
Mean	0.32	7.80	7.70	0.80	0.42	0.77
SD	0.12	1.62	2.50	0.10	0.26	0.09
CV	0.38	0.21	0.33	0.11	0.62	0.12

The numbers of allele per locus detected in this study were almost equivalent to those graded by Zehdi et al. (2004) who recognized 7.14 alleles per locus when examining 46 Tunisian date palm accessions using 14 microsatellite loci.

Elshibli and Korpelainen (2007) identified 21.4 alleles per locus, which were more than the numbers of alleles per locus detected in this study; it may be due to using more number of microsatellite loci (16) in addition to using different genotype area 68 Sudan and Morocco date palm accessions.

The ten primers used in this study successfully produced clear amplified SSR band sizes ranging from 108 bp (marker DP160) to 274 bp (marker DP175), similar to Ahmed and Al-Qaradawi (2009) results which ranged from 100–300 bp.

The sequences flanking microsatellite sites are generally conserved within species and also often in closely related species (Gupta and Varshney 2000). Interestingly, the ten SSR markers tested in this study formed mean of 7.8 genotype numbers (Table 2), however, the highest were 10 different genotypes scored in marker DP175 (206/262 bp, 198/198 bp, 236/274 bp, 196/196 bp, 200/258 bp, 198/234 bp, 226/226 bp, 196/236 bp, 226/250 bp and 234/270 bp) represented in Barhi, Hilali, Khalas, Khasab, Khnaizi, Lulu, Male, Razaiz, Shahil, and Shaishi, respectively while cultivar Sukari share the same genotype with cultivar Khnaizi.

On the other hand, only 4 different genotypes was renowned in marker DP186 (166/175 bp with Barhi, Hilali and Shaishi, 172/172 with Khalas, 163/163 with Khasab, Khnaizi, Lulu, Male, Shahil and Sukari, 163/172 bp with Razaiz cultivar.

The mean of gene diversity was 0.80 (Table 2) ranging from 0.60 (for locus DP168) to high diversity 0.90 (for two loci DP157 and DP175). This high level of gene diversity is similar to 0.83 reported in date palms germplasm from Sudan (Elshibli and Korpelainen 2007). This high level of diversity is expected because of the unique mechanism responsible for generating SSR allelic diversity by replication slippage. Replication slippage is thought to occur more frequently than single nucleotide mutations and insertion/deletion events, which generated the polymorphisms detected by RAPD analysis (Powell et al. 1996).

The heterozygosity was 0.42%, much lower than 0.84 and 0.82 in Sudan and Morocco date palm cultivars, respectively (Elshibli and Korpelainen 2007). The average major allele frequency was 0.32 ranging from 0.13 in marker DP175 to 0.59 in marker DP168. Similarly, the polymorphism information content PIC value which is commonly used in genetics as a measure of polymorphism for a marker locus used in linkage analysis, ranged between 0.55 in DP168 to 0.89 in DP157 and DP175, with an average of 0.77 per marker. Allelic variation might be correlated with the number of repeats within a particular microsatellite locus. A positive relationship was found between the number of repeats and the PIC of earlier reports in tomato (Smulders et al. 1997; Areshchenkova and Ganal 1999; He et al. 2003, Pritesh et al. 2010). In this study (Table 2), DP168 with the lower PIC (0.55) has eight repeats compared to DP157, which has PIC (0.89) with nineteen repeats, which is in agreement with the finding of Pritesh et al. (2010). Marker with lower PIC (0.08) has three repeats compared to marker which has PIC (0.40) with seven repeats. Moreover, similar to the report of He et al. (2003) no relationship was found between PIC and the number of nucleotides per repeat. However, there are reports that the polymorphism level in trinucleotide repeats is lower than that in di-nucleotide repeats for rice (Blair et al. 1999) and ryegrass (Jones et al. 2001).

**Fig. 2:**
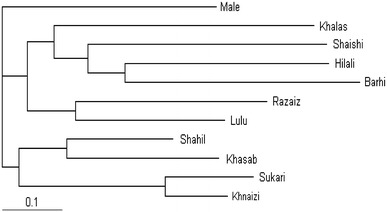
Phylogram Tree of Qatari date palm based on Distance of Nei 1983 by NJ method

A dissimilarity matrix between Qatari date palm cultivars showed an average dissimilarity coefficient ranging from 0.00 to 1.00. The cultivars studied here were highly divergent at the DNA level. The lowest dissimilarity coefficient value was observed between Khnaizi and Sukari cultivars (0.25) which seem to be the nearest two varieties and can be closely regrouped (Fig. 2). Ahmed and Al-Qaradawi (2009) found that the similarity coefficient value of Qatari date palm ranged from 0.00 to 0.75 while Zehdi et al. (2004) found that the similarity coefficient value of Tunisian date palm ranged from 0.3008 to 0.7885. The next closet cultivars with 0.43 dissimilarity coefficient value were obtained between Khasab and Shahil. Dissimilarity coefficient value of 1.00 was obtained between Barhi and each of Khasab, Khnaizi, Shahil and Sukarii cultivars, indicating that how far is the relationship between Barhi cultivar and those cultivars (Fig. 2). Molecular markers can provide an effective tool for efficient selection of desired agronomic traits because they are based on the plant genotypes and thus, are independent of environmental variation. Several molecular markers are being currently employed, of which simple sequence repeats (SSRs) or microsatellites are the most widely used types. SSRs are not only very common but also hypervariable for number of repetitive DNA motifs in the genomes of eukaryotes (Hamada et al. 1984; Edwards et al. 1991; Vosman and Arens 1997; Rallo et al. 2000; van der Schoot et al. 2000). All the other cultivars displayed different levels of dissimilarity but still were grouped with each others (Fig. 2). It is suggested that the variation or polymorphism of SSRs are a result of polymerase slippage during DNA replication or unequal crossing over (Levinson and Gutman 1987).

The phylogenetic diagram (Phylogram) illustrates the divergence between the studied Qatari date palm cultivars (Fig. 2) and suggests their tree branching. Male (Phahil) cultivar was clustered separately from the rest of other cultivars. Mainly the phylogenetic tree showed two major clusters, the first included four cultivars (Shahil, Khasab, Sukari and Khnaiz) and the second cluster contained six cultivars (Khalas, Shaishi, Hilali, Barhi, Razaiz and Lulu); however, Lulu and Razaiz were grouped in a separate sub cluster (Fig. 2).

Genetic diversity is desirable for long-term crop improvement and reduction of vulnerability in plants to important crop diseases. Measurements of genetic diversity can be used in breeding programs to increase the genetic variation in base populations by crossing cultivars with a high level of genetic distance as well as for the introgression of exotic germplasm. Molecular genetic diversity estimates are extremely useful for intellectual property protection, particularly in the determination of essential derivation. The genetic diversity estimates based on molecular marker data may be compared to a minimum genetic distance which indicates that two cultivars are not essentially derived (Lefebvre et al. 2001).
